# Video Assisted Anal Fistula Treatment in a Child with Perianal Fistula

**Published:** 2016-01-01

**Authors:** Naeem Liaqat, Asif Iqbal, Sajid Hameed Dar, Faheem Liaqat

**Affiliations:** Services Institute of Medical Sciences, Lahore, Pakistan

**Keywords:** Perianal fistula, VAAFT, Children

## Abstract

Perianal fistula formation is a rare complication in children after rectal biopsy. Perianal fistula may become difficult to treat; therefore a lot of surgical options are present. One of these options is video assisted anal fistula treatment (VAAFT). We present a 6-year-old female who developed perianal fistula following rectal biopsy for which VAAFT was done successfully.

## CASE REPORT

A 6-year-old female presented with discharge from gluteal region since three months. She was born through spontaneous vaginal delivery at a local hospital and had complaint of constipation since neonatal period. At the age of two years she was diagnosed as a case of hypothyroidism and thyroxin was started. She responded well to treatment and became euthyroid but her complaints of constipation did not resolve. Later on barium enema and rectal biopsy were advised and diagnosis of Hirschsprung’s disease was made. At the age of 3 year, Soave pull through was done. During subsequent 6 month she had repeated episodes of enterocolitis which were managed conservatively. Her complaints of constipation did not improve and repeat rectal biopsy showed aganglionosis. After three days of rectal biopsy, she presented with swelling at right gluteal region. Incision and drainage was done with suspicion of gluteal abscess but it turned out to be recto-cutaneous fistula. Trial of conservative management was given for 3 months but fistula did not heal. Examination of gluteal region showed two openings at 5 and 7’o clock position, 3 cm from anal verge (Fig. 1). It was labeled as high anal fistula and surgical treatment was advised. Video assisted anal fistula treatment was done in which fistuloscope was entered through the external opening of the tract. It was continuously being irrigated with normal saline. Whole of the tract was visualized and internal opening of the tract was located and ligated with suture on the mucosal side. Whole of fistula tract was cauterized and necrotic tissue excised. External opening was left open at the end of the procedure. Postoperatively the tract was daily flushed with normal saline through external opening. Gradually, the external opening healed by secondary intention. Patient is on follow up and is doing well with no recurrence (Fig 2).

**Figure:**
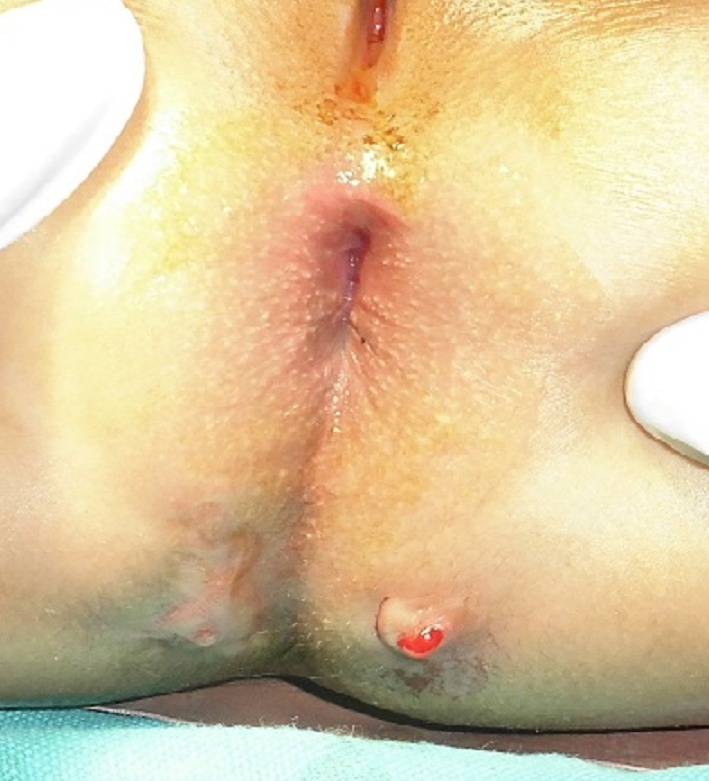
Figure 1:Perianal fistulae following rectal biopsy.

**Figure:**
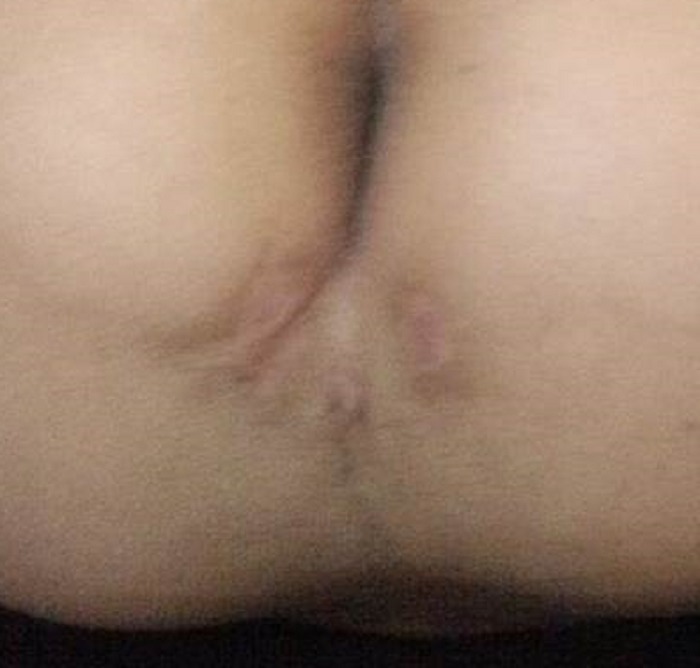
Figure 2:Post-operative picture showing healed fistulous tracts.

## DISCUSSION

Post-rectal biopsy anal fistula development is not a common complication. In order to deal with such an uncommon complication, particularly in a patient with previous soave pull through, only limited options were available to us. The options included Seton placement, anal plug treatment, VAAFT and redo pull through.[1] VAAFT is a novel surgical technique for treatment of complex anal fistulae without interfering with sphincter muscles. This technique includes two phases: a diagnostic and operative phase. In diagnostic phase, scope is passed from external opening to visualize whole of the tract and then internal opening of fistulous tract is localized. During therapeutic phase internal opening is closed, whole of the tract is cauterized and cyanoacrylate is injected at distal most part of the tract. The external opening is left open and tract is flushed for coming few days to heal spontaneously.[2,3]. Unfortunately all the treatment options for high anal fistula are associated with high incidence of recurrence and incontinence except VAAFT which has fairly less incidence of incontinence (5%).[4] In our case VAAFT was successful in managing complex perianal fistula.

## Footnotes

**Source of Support:** Nil

**Conflict of Interest:** None declared

